# Improvement of the affinity of an anti-rat P2X4 receptor antibody by introducing electrostatic interactions

**DOI:** 10.1038/s41598-021-03784-w

**Published:** 2022-01-07

**Authors:** Chinatsu Shinozaki, Keita Kohno, Mitsunori Shiroishi, Daisuke Takahashi, Yu Yoshikawa, Yoshito Abe, Kenji Hamase, Makoto Nakakido, Kohei Tsumoto, Kazuhide Inoue, Makoto Tsuda, Tadashi Ueda

**Affiliations:** 1 Department of Protein Structure, Function and Design, Graduate School of Pharmaceutical Sciences, Kyushu University, 3-1-1 Maidashi, Higashi-ku, Fukuoka, 812-8582 Japan; 2 Department of Molecular and System Pharmacology, Graduate School of Pharmaceutical Sciences, Kyushu University, 3-1-1 Maidashi, Higashi, Fukuoka 812-8582 Japan; 3 Department of Life Innovation, Graduate School of Pharmaceutical Sciences, Kyushu University, 3-1-1 Maidashi, Higashi, Fukuoka 812-8582 Japan; 4 Department of Drug Discovery and Evolution, Graduate School of Pharmaceutical Sciences, Kyushu University, 3-1-1 Maidashi, Higashi-ku, Fukuoka, 812-8582 Japan; 5 Department of Bioengineering, School of Engineering, The University of Tokyo, 7-3-1, Hongo, Bunkyo-ku, Tokyo, 113-8656 Japan; 6 Institute of Medical Science, The University of Tokyo, 4-6-1, Shirokanedai, Minato-ku, Tokyo, 108-8639 Japan; 7 Present Address: Department of Biological Science and Technology, Tokyo University of Science, 6-3-1 Niijuku Katsushika-ku, Tokyo, 125-8585 Japan

**Keywords:** Biophysics, Biotechnology, Neuroscience

## Abstract

We have recently developed a mouse monoclonal antibody (12–10H) binding to the head domain region in rat P2X4 receptor (rP2X4R, which is crucial for the pathogenesis of neuropathic pain) expressed on the cell with the highest binding affinity (*K*_D_ = 20 nM). However, the 12–10H antibody failed to detect endogenously expressed P2X4Rs in microglia isolated from the spinal cord of rats whose spinal nerves were injured. Then, we prepared R5 mutant, in which five arginine residues were introduced into variable regions except for the “hot spot” in the 12–10H antibody to increase electrostatic interactions with the head domain, an anionic region, in rP2X4R. The mutation resulted in an increase of 50-fold in the affinity of the R5 mutant for the head domain with respect to the intact 12–10H antibody. As a result, detection of P2X4Rs endogenously expressed on primary cultured microglial cells originated from the neonatal rat brain and spinal cord microglia isolated from a rat model of neuropathic pain was achieved. These findings suggest a strategy to improve the affinity of a monoclonal antibody for an anionic antigen by the introduction of several arginine residues into variable regions other than the “hot spot” in the paratope.

## Introduction

P2X receptors are ATP-activated trimeric ion channels widely expressed in the nervous system and peripheral organs implicated in various functions under the healthy and disease conditions^1–3^. The subtype, P2X4 receptor (P2X4R) has been implicated in neuropathic pain that occurs after damage to the nervous system resulting from compression of cancer, diabetes, viral infections, chemotherapy, or traumatic injury^4,5^. In animal models of neuropathic pain, the expression of P2X4R is upregulated selectively in microglia in the spinal cord, and pharmacological blockade and genetic knockout of P2X4R suppress behavioral pain hypersensitivity^6–8^. Mechanistically, extracellular ATP binds to P2X4Rs expressed on the surface of spinal cord microglia, which in turn causes depolarization and an influx of Ca^2+^ and a release of diffusible factors including brain-derived neurotrophic factor, resulting in altering nociceptive neurotransmission^6,9,10^. In the crystal structure of P2X4R, the receptor subunit adopts a dolphin-shaped structure comprising an extracellular head, a body, and a fluke domain^11^. When ATP binds, the movement of these domains modulates the conformational change of the trimer structure and subsequently gives rise to the opening and closing of pores causing Ca^2+^ influx^11,12^. The development of antibody against the extracellular domains (head, upper body, and lower body domains) of P2X4R could be useful not only for identification of P2X4R expression on cell surface, but also for assessing its expression levels, which might open an avenue to develop a new diagnostic marker for neuropathic pain.

In our previous study, by expressing the head domain of rat P2X4R (rHD, Gln111–Val167) in *E. coli*, we showed an intact structure with three correctly formed S–S bonds^13^ and developed five anti-rHD monoclonal antibodies that recognize the conformational epitope on the rHD. Among these antibodies, the monoclonal antibody 12–10H showed the highest binding affinity and detected rat P2X4R (rP2X4R) expressed on the cells^14^. Since we have shown the paratope of the 12–10H monoclonal antibody, with the highest binding affinity to rHD (*K*_D_ = 20 nM)^14^ at the present, we will be able to improve the affinity of 12–10H to rHD by introducing mutations except for “hot spot” of the 12–10H antibody. In this study, we developed an engineered monoclonal antibody with 50 times greater binding affinity to rHD than that of the intact 12–10H antibody. Moreover, we demonstrated that the engineered antibody bound rP2X4Rs expressed on the cell not only in primary culture microglia but also in vivo samples in comparison with the negative control antibody, in which Tyr 33 and Arg52 in the heavy chain and Tyr31 in light chain in 12–10H were mutated to Ala, simultaneously, prepared based on our recent study^15^.

## Results

### Flow cytometry analysis of fluorescent labeled anti-rP2X4R antibody (12–10H) to detect rP2XR on living cells

**Figure 1:**
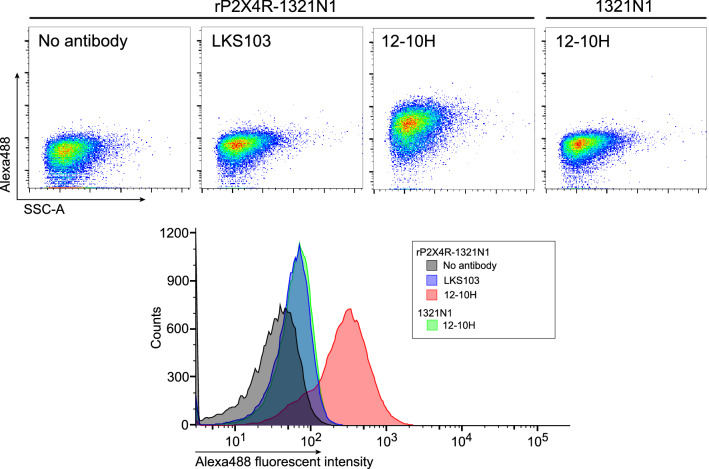
Staining of rat P2X4Rs expressed on surface of living cells by fluorescent-labeled 12–10H antibody. Flow cytometry pseudocoloured scatterplots (upper panels) and histograms (lower panel) of living 1321N1 cells with or without P2X4R expression. These cells were stained with or without Alexa Fluor 488-labeled 12–10H or LKS103 antibody (40 μg/ml) for 30 min at 37 °C.

We have recently developed the monoclonal antibody 12–10H with very high affinity to rHD (*K*_D_ = 20 nM) and use it to detect the P2X4Rs stably expressed in 1321N1 human astrocytoma cells (P2X4R-1321N1 cells)^14^. In this study, we conjugated the 12–10H antibody with Alexa Fluor 488 (the degree of labeling (DOL) was 2.1, indicating that one antibody molecule was labelled with two fluorescent dyes) and examined the ability of the fluorescent labeled 12–10H antibody to bind to rP2X4Rs. As a negative control, an antibody for hen egg lysozyme (LKS103), has been already developed in our laboratory, that had been similarly labeled by Alexa Fluor 488 (DOL: 2.4) was used. The fluorescent labeled 12–10H antibody resulted in an increase of the fluorescent intensity of P2X4R-1321N1 cells compared with no antibody treatment (Fig. 1). The increase in fluorescence in antibody treated 1321N1 cells lacking P2X4R expression was not observed. In addition, the fluorescence intensity in P2X4R-1321N1 cells was not affected when treated with the negative control Alexa Fluor 488-labeled LKS103 antibody. These results indicate that the fluorescent labeled 12–10H antibody detects P2X4Rs expressed on living cells. However, the detection by this antibody is not quantitative, since fractions of 1321N1 cells treated with and without the antibody partly overlapped. To separate these fractions more clearly, we increased the amount of Alexa Fluor 488 conjugated to 12–10H antibody (DOL: 5.3). Compared with 12–10H with normal labeling (DOL: 2.1), the distribution of P2X4R-expressing 1321N1 cells detected by highly labeled 12–10H antibody was skewed to the right (Fig. S1), implying a slight improvement of the separation. When we also examined the influence of Alexa Fluor 488 labeling on binding affinity of 12–10H antibody to rHD using surface plasmon resonance (SPR) (Fig. S2), it was confirmed that dissociation constant (*K*_D_) of the two 12–10H antibodies (with normal and high labeling) to rHD complex was similar (Fig. S2). This was consistent with our structural model showing that lysine residues are not located in the complementary determining region of 12–10H as was shown in previous study^15^. From these results, it was suggested that the 12–10H antibody did bind to rP2X4Rs expressed in 1321N1 cells but, for improved detection of rP2X4Rs, its binding affinity needed to be further enhanced.

### Design and preparation of the high affinity R5 mutant

**Figure 2:**
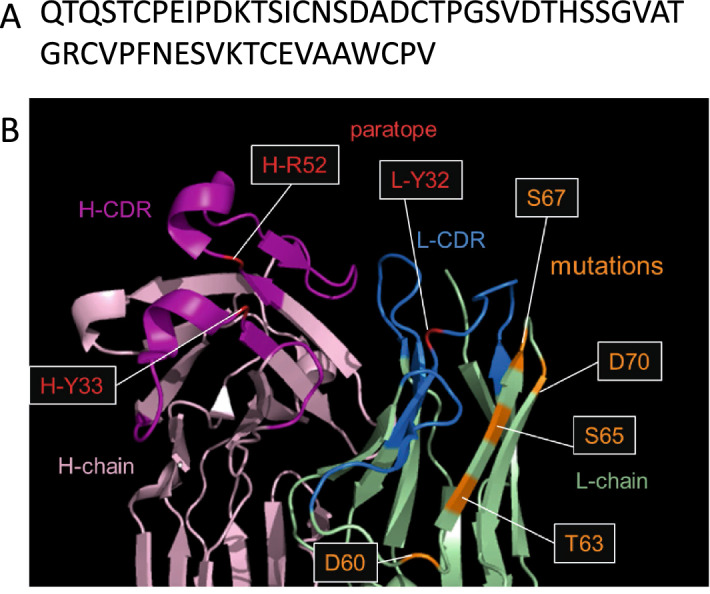
Homology model of the 12–10H antibody. (**A**) Primary sequence of the head domain (Gln111–Val167) in rP2X4R. (**B**) The location of mutated site in the model of 12–10H Fab based on the crystal structure of CD147 C2 domain in the complex with Fab of its monoclonal antibody 6H8 (PBD id 5X0T).

Antigen–antibody interactions are orchestrated by complementarity in the charge and shape of the epitope (in an antigen) and paratope (in an antibody) and are of critical importance for protein engineering. In our previous studies^16,17^, we have targeted the framework regions (FR, region other than the paratope) of light chain for protein engineering to increase the affinity of antibodies. Since the FR is highly conservative and highly homologous among antibodies of the same species, improving the affinity by adding mutations in the FR of light chain could be a versatile approach for engineering other antibodies. Indeed, we have succeeded in preparation of the antibody with high binding affinity for anionic antigens such as human cardiac troponin I^16^ and insulin^17^ by introducing several Arg residues in the FR of antibody light chains, to enhance long range electrostatic interactions^16,17^. As is the case with troponin and insulin, rHD is an anionic antigen where acidic residues are located on the surface of the protein^13^, with three disulfide bonds (Fig. 2A). We have also demonstrated in our previous study that the epitope in rHD consists of Asn127 and Asp131. Furthermore, our recent report has determined the complementarity determining regions (CDRs) in 12–10H in the antigen-binding fragment (Fab) from 12–10H antibody and shown that the paratopes are composed of Y33, R52, E58 in Fab heavy chain and Y32 in Fab light chain^15^. In these contexts, we have attempted the construction of the 12–10H Fab mutants for increased affinity toward rHD. According to the methodology of our previous studies^16,17^, we targeted the FR of light chain, and constructed the mutant Fab where Asp60, Thr63, Ser65, Ser67 and Asp70 in the 12–10H light chain were replaced with Arg, simultaneously (denoted R5 mutant) (Fig. 2B).

**Figure 3:**
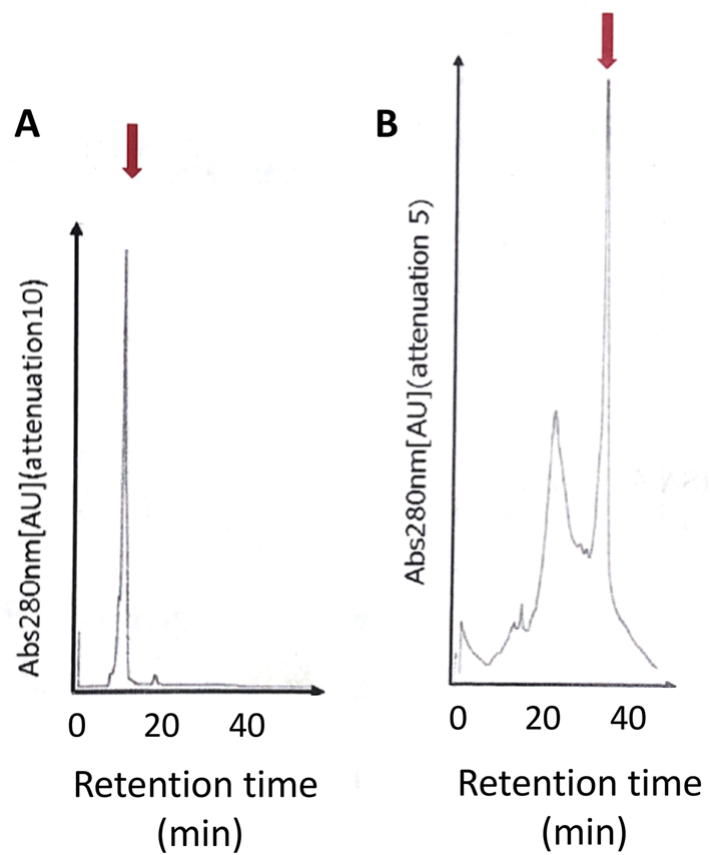
Fab preparation. Ion exchange of HPLC pattern of the refolded Fab from 12–10H (**A**) and the refolded Fab from R5 mutant (**B**). The column (Resource S) was eluted with a gradient of 1 M NaCl at a flow rate of 1 ml/min.

Since the theoretical pI of heavy chain of Fab from 12 to 10H is 8.9 and that of light chain is 5.2, it is expected that the introduction of positive charge to light chain would increase the solubility during purification by cation-exchange at pH 4. The Fab region of the heavy chain of 12–10H, light chain of 12–10H and light chain of R5 mutant Fab were expressed in *E.coli*, and the crude extracts were cleared from nucleic acids as described in material and methods. This material was then reduced by 2-mercaptoethanol and incubated with 3-trimethylammoniopropyl methanethiosulfonate (TAPS-sulfonate), which reversibly reacts with the thiol groups to give protein of strong positive charge^18^. We found that these fragments were retained on cation-exchange equilibrated with 50 mM acetate buffer at pH 4 and eluted with a 0 to 1 M NaCl gradient, with a slight modification of our previous report^19^. Each protein was eluted as major peak as monitored at 280 nm. SDS-PAGE showed a single band with a molecular weight between the molecular weight marker of 21.5 kDa and 44.3 kDa from each construct (data not shown). Refolding of Fab originated from 12–10H or R5 mutant using individual heavy chain and light chain of Fabs was conducted according to the methodology described in our previous research^15,19^. The separation profiles of refolded Fabs by cation exchange chromatography are shown in Fig. 3. As predicted from the increase content of positive charge, the refolded R5 mutant Fab eluted later than the refolded 12–10H Fab. We also confirmed by SDS-PAGE that the peaks indicated by an arrow in Fig. 3A and B contained the proteins (Fig. 3S). These results suggested that R5 mutant Fab was properly refolded as well as Fab from 12 to 10H.

### Evaluation of binding affinity of Fab from R5 mutant for rHD

**Figure 4:**
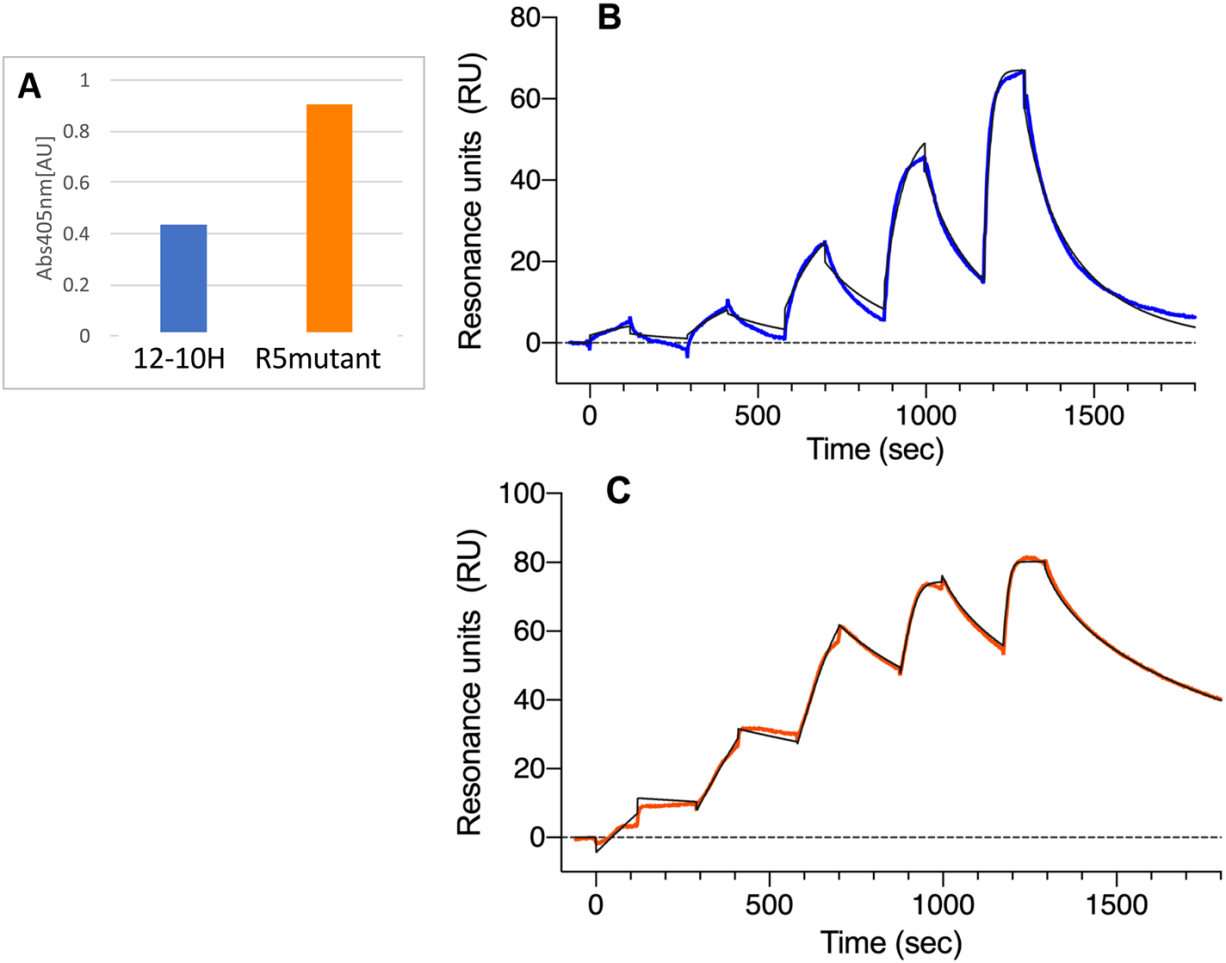
Binding analysis of antibodies to rHD. (**A**) ELISA analysis using the refolded Fab from 12–10H (colored in blue) and R5 mutant (colored in orange) as a primary antibody and alkaline phosphatase-conjugated anti-mouse Fab-specific antibody as a secondary antibody at 37 °C. B. Kinetic analysis of the interaction between 12–10H and rHD at 25 °C. (**C**) Kinetic analysis of the interaction between R5 mutant and rHD at 25 °C. SPR sensorgrams were fitted by global analysis (black lines) using the BIAevaluation software.

At first, we compared the binding affinity of Fab from R5 mutant for rHD with that of Fab from 12–10H using ELISA. The results indicated that the Fab from the R5 mutant had a higher affinity to rHD than that from 12–10H (Fig. 4A).

We next evaluated the kinetic parameters of the interaction between rHD and 12–10H Fab or R5 mutant Fab using surface plasmon resonance (SPR) (Fig. 4B and C). The *K*_D_ of these Fabs for rHD at 25 °C are given in Table 1. The *K*_D_ of Fab from 12 to 10H-rHD complex was 9.4 nM that was almost identical to that described in our previous study^15^. The binding affinity of the R5 mutant Fab for rHD was about 50 times higher than that of Fab from 12–10H due to both faster association phase (*k*_*on*_) and slower dissociation step (*k*_*off*_).

**Table 1:** Dissociation constants of Fab-rHD interactions at 25 °C.

Analyte	*K*_D_ (nM)	*k*_*on*_ (M^−1^ s^−1^)	*k*_*off*_ (s^−1^)
12–10H	12.34 ± 3.67	(1.03 ± 0.31) × 10^6^	(1.20 ± 0.11) × 10^−2^
R5 mutant	0.25 ± 0.02	(1.16 ± 0.45) × 10^7^	(2.93 ± 0.11) × 10^−3^

### In vitro and in vivo binding assay of R5 mutant antibody for the detection of rP2X4Rs expressed on cells

**Figure 5:**
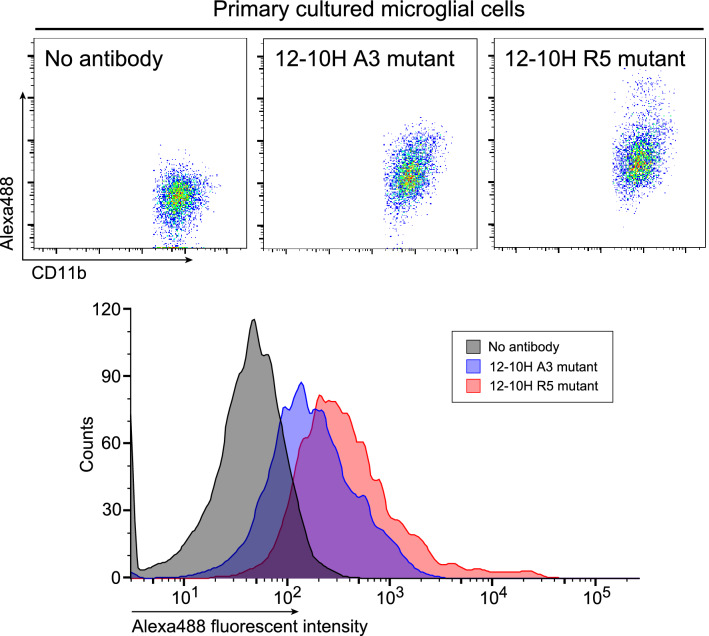
Detection of P2X4Rs expressed on surface of living rat microglial cells by 12–10H R5 mutant antibody. Flow cytometry pseudocoloured scatterplots (upper panels) and histograms (lower panel) of living primary cultured microglial cells. Cells were stained with or without Alexa Fluor 488-labeled 12–10H A3 or R5 mutant antibody (40 μg/ml) for 30 min at 37 °C.

**Figure 6:**
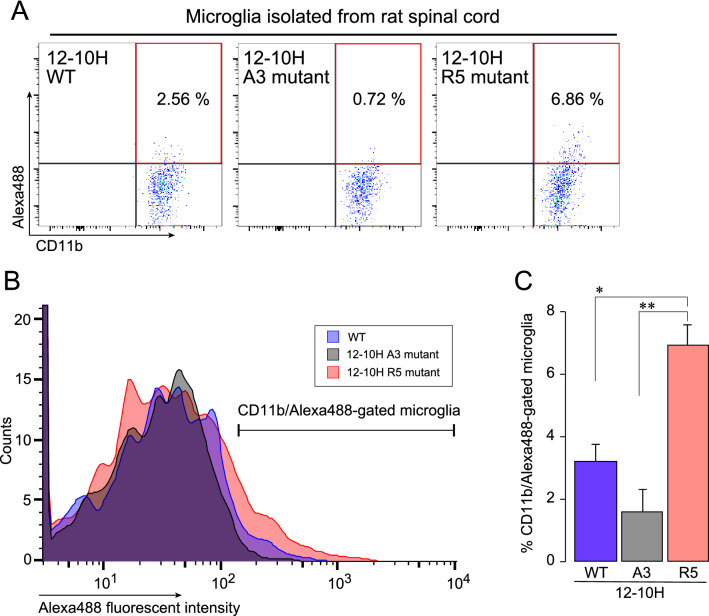
12–10H R5 mutant antibody staining of P2X4Rs on living spinal cord microglia isolated from a rat model of neuropathic pain. (**A**) Flow cytometry pseudocoloured scatterplots of living microglia isolated from the spinal cord tissue of rats on day 14 after spinal nerve injury. Isolated living cells were stained with or without Alexa Fluor 488-labeled 12–10H WT, A3 or R5 mutant antibody (40 μg/ml) for 30 min at 37 °C. Numbers indicate percentage of CD11b and Alexa Fluor 488-gated cells (red square). (**B**) Histograms (lower panel) show overlay of P2X4R expression on spinal cord microglia, gated as in A. (**C**) Percentage of CD11b/Alexa488-gated spinal cord microglia (shown in A) (n = 3 rats). *P < 0.05 and **P < 0.01, one-way ANOVA with post hoc Tukey’s multiple comparison test. Data are the mean ± SEM.

To detect endogenously expressed rP2X4Rs, we used primary cultured microglial cells prepared from neonatal rats and analyzed the fluorescence intensity of the antibody-labeled cells by flow cytometry. The intensity level of Alexa Fluor 488 was increased in microglial cells treated with R5 mutant antibody compared with A3 mutant antibody (Fig. 5), suggesting that R5 mutation enhances the ability of the 12–10H antibody to detect endogenous rP2X4Rs on living microglial cells. We further tested this antibody using microglial cells isolated from the rat spinal cord tissue on day 14 after nerve injury, a model of neuropathic pain whose spinal cord microglia upregulate P2X4R expression (Fig. 6). The number of spinal cord microglia in the range of high fluorescent intensity of Alexa Fluor 488 was significantly higher in the R5 mutant antibody than others (Fig. 6). These results indicate that the R5 mutation improves the capability of the antibody to detect rP2X4Rs endogenously expressed on plasma membrane of activated spinal cord microglia in a model of neuropathic pain.

## Discussion

Despite recent advances in the understanding of the role of P2X4Rs and microglia as key players in the pathogenesis of neuropathic pain^20^, pharmacological tools for P2X4R are still underdeveloped^21^. Therefore, the monoclonal antibody against P2X4Rs was prepared by use of gene gun immunization, followed by a boost using transfected cells expressing P2XRs and the resulting monoclonal antibodies were characterized^22^. Moreover, structural and function studies of the P2X4Rs using monoclonal antibodies have been reviewed^23^. In this context, with the aim of detecting the rP2X4Rs expressed cells in vitro and in vivo, we report the preparation of an engineered antibody with high affinity to rP2X4R and characterization of the resulting mutant antibodies.

At first, we tried to detect rP2X4R expressed on 1321N1 cells using the fluorescent labeled 12–10 H antibody (Fig. 1) and the highly fluorescent labelled 12–10H (Fig. S1), but the fractions of 1321N1 cells treated with and without the antibody still partly overlapped. To improve the detection of rP2X4R expressed on the cells, it was necessary to prepare an engineered antibody with much higher affinity to rP2X4R. Previously, we have succeeded to increase of the binding affinity of single-chain variable fragment (scFv) to human cardiac troponin I^16^ and that of Fab to human insulin^17^ more than 100 times than that of respective intact scFv and Fab by mutating five residues of the FR region of light chain into arginine. Since these antigens were anionic proteins like rHD, we expected that the introduction of five residues of arginine into the framework region of Fab in 12–10H would enhance the affinity to rHD as well in Fig. 2B. Moreover, since we have determined the hot spots (Tyr 33, Arg52 in heavy chain and Try32 in light chain) in 12–10H to rHD^15^, we could confidently design R5 mutation into the region except for paratope of Fab in 12–10H (Fig. 2B). As a result, we showed that R5 mutant Fab had high binding affinity to rHD in the sub-nanomolar range, whereas Fab from 12–10H displaced an affinity of ~ 12 nM (Table 1). Then, we prepared R5 mutant antibody and A3 mutant antibody (as a negative control) by employing the CHO expression system to detect rP2X4Rs expressed on primary cultured microglial cells originated from the neonatal rat brain and spinal cord microglia isolated from a rat model of neuropathic pain. In the near future, we may understand a part of the functions of rP2X4R and its-expressing microglia using R5 mutant antibody.

Head domain of zebrafish P2X4R is exposed to the solvent^11,12^. According to sequence similarity, rP2XR is also expected to be exposed to the solvent. This is consistent with that we could prepare the antibody, bound to rP2X4Rs expressed on the cells, by immunization to mice of rHD^14^. Sequence alignment of the head domain of rat P2X1, P2X2, P2X3, P2X5, P2X6 and P2X7R based on the sequence of rHD of P2X4R (colored in red) is shown in Fig. 4S. These head domain of rat P2X1R, P2X2R, P2X3R, P2X5R, P2X6R and P2X7R will be exposed based on sequence similarity. Since the head domains of rat P2X1R, P2X2R, P2X3R, P2X5R and P2X6R are predicted to be anionic, the present findings may contribute to generate antibodies with high affinity bound to head domains of each individual class of receptor. So far, there have been a significant progress for the function of P2X7R in microglia. Since the head domain of P2X7R is rather cationic (predicted pI is 8.6) compared to the one of P2X4R (4.4), we believe that the protein engineering that introduces cationic residues into the 12–10H antibody may contribute to increasing the selectivity between P2X4R and P2X7R. Additionally, the methodology described in this study may be enlarged to improve the binding affinity of an antibody recognizing anionic antigens.

## Methods

### Flowcytometry

1321N1 human astrocytoma cells stably expressing rP2X4Rs^14^ were maintained in Dulbecco’s-modified Eagle’s medium (DMEM) supplemented with 10% fetal bovine serum (FBS) in a humidified atmosphere of 5% CO_2_ at 37 °C. Cells were detached by exposing to 0.05% trypsin–EDTA and collected by pipetting. After washing with Hanks' balanced salt solution containing 2% FBS (HBSS-FBS), cells were incubated with the pre-warmed antibodies (40 μg/ml) for 30 min at 37 °C. After washing, the pellet was resuspended in ice-cold HBSS-FBS and filtered through a 35-μm nylon cell strainer (BD Biosciences). The antibody binding was detected using a channel for FITC of FACSVerse (BD Biosciences).

### Expression heavy chain and light chain from 12–10H and light chain from R5 mutant

A DNA sequence encoding heavy chain in Fab region and light chain from 12–10H, and light chain from 5 mutant was inserted into pET22a vector, and transformed into *Escherichia coli* strain BL21(DE3) codon plus RIL cells (Novagen, Madison, WI, USA), respectively. The transformant cell was grown at 37 °C in 1 L of LB medium containing 50 μg/ml ampicillin. The culture was allowed to grow until mid-log phase (OD600 = 0.6–0.8) and protein expression was induced with 1 mM isopropyl-beta-D-thiogalactopyranoside (IPTG) for 4 h at 37 °C.

### Purification of each heavy chain and light chain from 12–10H and light chain from R5 mutant from inclusion body

Antibody heavy chain in Fab region and light chain from 12–10H were purified, respectively, as described in our previous study^19^. The cultured cells were harvested by centrifugation for 10 min at 8000 rpm. The insoluble fraction was suspended in 20 ml of 20 mM MOPS buffer, pH 7, and then sonicated 3 times for 3 min in an ice-water bath. The mixture was centrifuged for 60 min at 12,000 rpm, 4 °C. The precipitates were resuspended in 10 ml of 20 mM MOPS buffer, pH 7. DNase I (final concentration of 5 μg/ml), RNase A (final concentration of 20 μg/ml) and MgCl_2_ (final concentration of 10 mM) were added into the mixture to degrade nucleic acids. The mixture was incubated at 40 °C for 1 h followed by centrifugation. The collected pellets was washed with 25 ml of 0.8 M sodium chloride and centrifuged. This washing step was repeated 3 times. The insoluble material was next dissolved in 20 ml of 6 M guanidine solution (0.584 M Tris–HCl buffer, pH 8.6, containing 5.37 mM EDTA and 6 M guanidine hydrochloride) and reduced with 50 μl of mercaptoethanol at 40 °C for 90 min under a nitrogen atmosphere. TAPS sulfonate (225 mg), which reversibly reacts with the thiol groups to give protein strong positive charge, was added, and the reduction solution was incubated at 40 °C for 30 min to increase the solubility of the denatured Fab fragments. The reaction mixture was dialyzed against 50 mM sodium acetate buffer, pH 5.5, containing 8 M urea and passed through an anion exchange (DEAE-Toyopearl) column (1.5 cm × 20 cm) equilibrated with 50 mM sodium acetate buffer at pH 5.5, containing 8 M urea to remove the residues of nucleic acids. The flow-through solution was applied to a cation-exchange (SP-Toyopearl) column (1.5 cm × 30 cm) equilibrated with 50 mM sodium acetate buffer at pH 5.5. The column was eluted with NaCl gradient (0–1 M). The protein fraction was collected and lyophilized.

### Folding and purification of Fab from 12–10H and from R5 mutant

The folding of Fab was carried out using the dialysis method according to the method of Fujii et al.^19^ with slight modification. The lyophilized TAPS heavy chain and light chain from 12–10H or the lyophilized TAPS heavy chain from 12–10H and light chain from R5 mutant were separately dissolved in 3 ml of 6 M guanidine solution, respectively. Each fragment solution (82 nmol) were quantified by the absorption at 280 nm and then were mixed in 1:1 molar ratio. Cysteamine (final concentration of 72 mM) was added to the mixed solution of heavy chain and light chain from 12–10H or the solution of heavy chain from 12–10H and light chain from R5 mutant for the reduction. After incubation at 40 °C for 90 min, cystamine (final concentration of 26 mM) was added to the respective reduced solution (redox solution). To the redox solutions, 3 ml of the dialysis buffer (0.1 M Tris–HCl buffer, 1 mM EDTA at pH 8.0 containing 0.3 mM cysteamine, and 0.3 mM cystamine) in the presence of 8 M urea were added respectively (protein concentrations are 30 μM), and the diluted solution was dialyzed twice against the dialysis buffer (0.1 M Tris–HCl buffer, 1 mM EDTA at pH 8.0 containing 0.3 mM cysteamine and 0.3 mM cystamine) in the presence of 8 M urea for 4 h. Then, the dialysis was performed against the dialysis buffer in the presence of 4, 2, 1, and 0 M urea. Finally, the dialysis was performed against 0.1 M buffer containing 1 mM EDTA, at pH 8) for 100 h. The separation of folded Fabs were conducted by ion-exchange HPLC. The refolded Fab from 12–10H and that from R5 mutant were applied to the column of Resource S (1 ml, cytiva) equilibrated with 30 mM MOPS-NaOH at pH 7.0, respectively. NaCl gradient (a 0–1 M) was applied at a flow rate of 1 ml/min and the elution was monitored by absorbance at 280 nm.

### Preparations of mutant 12–10 H antibodies by CHO expression system

R5 mutant antibody where Asp60, Thr63, Ser65, Ser67 and Asp70 of the 12–10H light chain were replaced with Arg, simultaneously, and A3 mutant antibody where Tyr33 and Arg52 in the heavy chain and Tyr32 in the light chain of 12–10H were mutated with Ala, simultaneously, were prepared by CHO expression system following the methodology described previously^24^. In principle, the DNA sequences of the heavy and light chains of the antibody were subcloned into the pcDNA3.4 vector (Thermo Fisher Scientific). The vectors were transiently transfected into ExpiCHO cells (Thermo Fisher Scientific) using ExpiFectamine CHO Transfection Kit (Thermo Fisher Scientific) in accordance with the manufacturer's standard protocol. The cells were cultured for 8 days at 37 °C and 8% CO_2_. The cultures were centrifuged at 400 × g for 15 min, and the supernatant was collected. Each supernatant was applied onto an rProtein A Sepharose Fast Flow column (GE Healthcare) equilibrated with PBS at pH 7.4. The fraction bound to the column was washed with the PBS and subsequently eluted with Pierce IgG Elution Buffer (Thermo Fisher Scientific). The eluted fraction was neutralized by addition of 2 M Tris–HCl (pH 8.0) and further purified by size exclusion chromatography using a HiLoad 16/600 Superdex 200 pg column (GE Healthcare) equilibrated with PBS at pH 7.4.

### ELISA

ELISA was carried out as described in our previous report^14^. A 96-well immunoplate was coated with 100 μl of 1 μM rHD in carbonate buffer at pH 9.6 for 1 h. The plate was blocked by phosphate-buffered saline (PBS) containing 5% skim milk (Nakalai Tesque) and 0.005% Tween 20 for 1 h to reduce nonspecific adsorption. Then, the plate was washed with PBS containing 0.005% Tween 20 (PBST) and reacted with 100 μl of the prepared monoclonal antibody dissolved in PBS for 1 h, followed by washing with PBST three times. The bound antibody was reacted with 100 μl of 10,000-fold diluted alkaline phosphatase–conjugated goat anti-mouse IgG (Abcam, Cambridge, UK) for 1 h. After the plate was washed three times with PBST, alkaline phosphatase conjugation substrate dissolved in AP color developing buffer (BIO-RAD, San Francisco, USA), was added to each well and incubated for 15 min. Absorbance at 405 nm was measured with a Multiskan FC plate reader (Thermo Scientific).

### Labeling of monoclonal antibodies with succinimidyl ester of Alexa Fluor 488

The 12–10 H antibodies were labeled with Alexa Fluor 488 (Thermo Fisher scientific) at amino group, according to the manufacturer’s instruction. After dialysis of purified antibodies against phosphate-buffered saline (PBS; pH 7.4) at 4 °C, one-tenth amount of 1 M sodium bicarbonate solution was added to the antibody solution. Alexa Fluor 488 dissolved in anhydrous dimethyl sulfoxide (10 mg/ml) was added to the antibody solution at a dye/protein molar ratio of 1 ~ 20. The reaction mixture was stirred overnight at room temperature in the dark followed by the addition of ethanolamine at a final concentration of 100 mM. Unconjugated dye was removed by dialysis against PBS at 4 °C. The dye/protein ratio in the Alexa Fluor 488-labeled antibody was determined by absorbance measurements at 280 and 494 nm. Labeled antibodies were kept at 4 °C in the dark until use.

### Surface plasmon resonance

The kinetic parameters for interactions between the anti-rP2X4 antibody and rHD mutants were measured by SPR using a Biacore X100 instrument (GE Healthcare) with a running buffer of 10 mM HEPES buffer (pH 7.4) containing 150 mM NaCl, 0.05 mM EDTA, and 0.005% (v/v) Tween 20. The rHD was immobilized on a CM5 chip by amine coupling chemistry. Serial dilutions of purified 12–10H Fab or 12–10 Fab R5 mutant were injected into the rHD-immobilized and blank channels (for reference subtraction) at a flow rate of 30 μl/min, with an association time of 100 s and a dissociation time of 100 s. Kinetic parameters were calculated by fitting to a 1:1 (Langmuir) binding model using Biacore Evaluation Software (GE Healthcare). To assess the effect of Alexa Fluor 488 labeling on binding affinity of 12–10 H to rHD, the intact 12–10H IgG or Alexa Fluor 488-labeled 12–10H IgG was immobilized on a CM5 sensor chip via amine coupling. Purified rHD was then injected at a series of concentration in the running buffer at a flow rate of 30 μl/min.

### Primary cultured microglial cells

Primary cultured microglial cells were prepared according to a previously described method^25,26^. 1321N1 human astrocytoma cell lines were a gift from Dr. Michael W. Salter, The Hospital for Sick Children, Toronto, Canada. In brief, a mixed glial culture was prepared from neonatal Wistar rats (CLEA Japan) and maintained for 13 days in DMEM with 10% FBS. Immediately before experiments microglia were collected as the floating cells over the mixed glial culture by a gentle shake of the culture flasks. The collected cells were centrifuged and resuspended with FBS-HBSS. The cell suspension was blocked by incubating with Fc Block (anti-rat CD32, BD Biosciences) for 5 min at 4 °C and immunostained with the antibodies (3A or R5 mutant) and CD11b-Alexa Fluor 647 (BD Biosciences) for 30 min at 37 °C. After washing, the cells were analyzed as described above.

### Microglia isolated from spinal cord tissue

Methods employed in this study are are reported in accordance with ARRIVE guidelines (https://arriveguidelines.org).

Male Wistar rats (CLEA Japan) were aged 9 weeks at the start of the experiment and were housed in individual cage at a temperature of 22 ± 1 °C with a 12/12 h light/dark cycle (lights on 8 A.M. to 8 P.M.), and fed food and water ad libitum. All animal experiments were conducted according to the national and international guidelines contained in the Act on Welfare and Management of Animals (Ministry of Environment of Japan) and Regulation of Laboratory Animals (Kyushu University) and under the protocols approved by the Institutional Animal Care and Use committee review panels at Kyushu University. To develop a model of neuropathic pain^27,28^, the left fifth lumbar (L5) spinal nerve was tightly ligated with 5–0 silk and cut just distal to the ligature under isoflurane (2%) anesthesia. The wound and the surrounding skin were sutured with 3–0 silk. Fourteen days later, rats were deeply anesthetized by *i.p.* injection of pentobarbital and perfused transcardially with PBS, and the fourth and fifth lumbar segments of the spinal cord ipsilateral to the injury was isolated. Spinal tissue pieces were treated with pre-warmed 0.8 ml enzymatic solution [0.2 U/ml collagenase D (Roche) and 4.3 U/ml of dispase (GIBCO)] in HBSS-FBS for 30 min at 37 °C. The tissues were homogenized by passing through a 23G needle attached with a 1 ml syringe and were further incubated for 15 min at 37 °C. After that, the tissues were homogenized by passing twice through a 26G needle, and the enzymatic reaction was stopped by adding EDTA (0.5 M). Myelin debris was removed from the cell suspension using Myelin Removal Beads II and a MACS LS column (Miltenyi Biotec, Bergisch-Gladbach, Germany) according to the manufacturer's protocol. After centrifugation, the cells were resuspended in HBSS-FBS. The suspension blocked with Fc Block were immunostained and analyzed by flow cytometry as described above.

## Supplementary Information

Supplementary Information.
