# Supplementary material for: Improvement of the affinity of an anti-rat P2X4 receptor antibody by introducing electrostatic interactions
Source: Sci Rep. 2022 Jan 7;12:131. doi: 10.1038/s41598-021-03784-w (PMC8742113; doi:10.1038/s41598-021-03784-w)
Supplement: Supplementary file 1 — Supplementary Information. [file 41598_2021_3784_MOESM1_ESM.pdf]

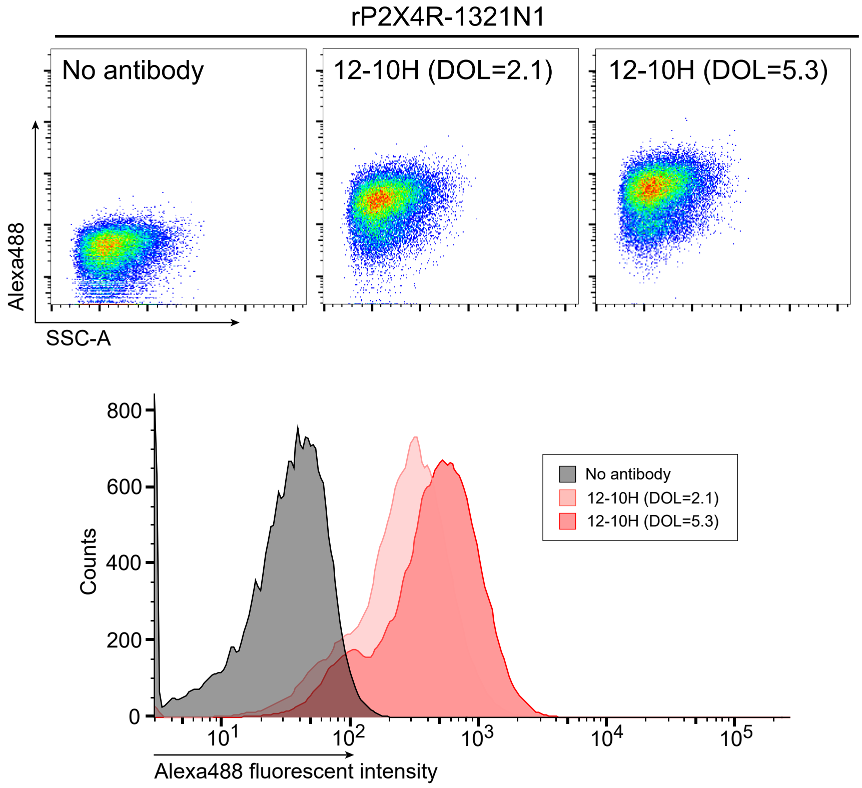

Shinozaki et al. Figure S1

A

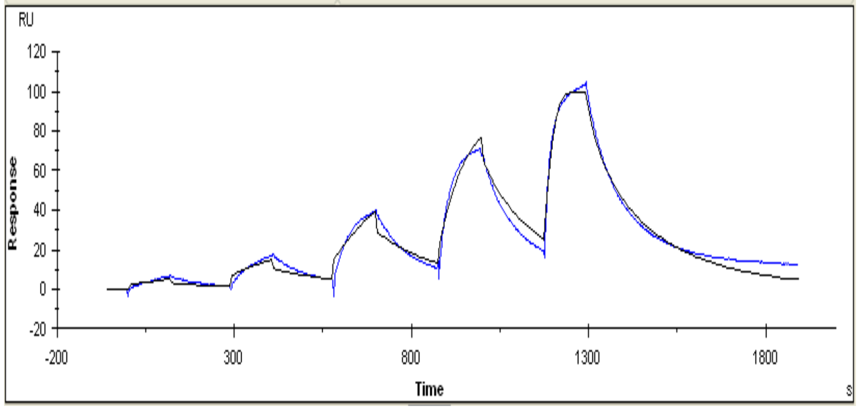

kon(1/Ms)	koff(1/s)	K_D(nM)
1.974E+5	0.01620	82.1

B

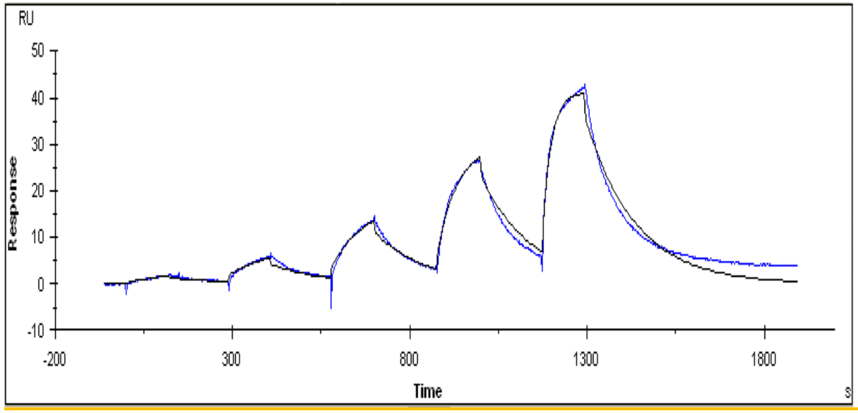

kon(1/Ms)	koff(1/s)	K_D(nM)
6.470E+4	0.007149	110.5

Shinozaki et al. Figure S2

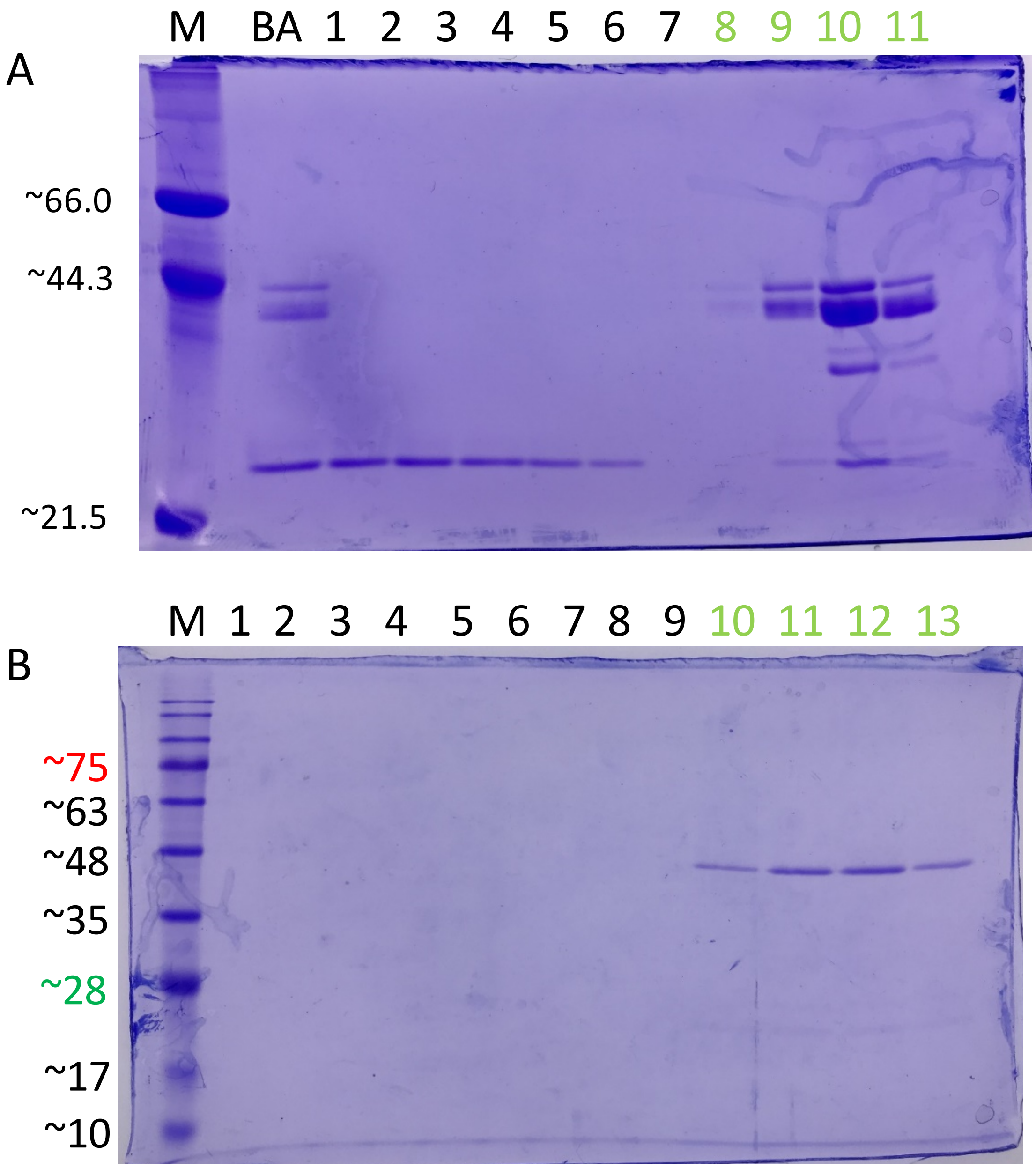

M, marker
BA, before applying column
8-11, elution fractions

M, marker,
10-13, elution fractions

Sinozaki et a. Figure S3

```
     111      120        130       140       150        160    167
P2X4 QTQSTCPEIPDKTS-ICNSDADCTPGSVDTHSSGVATGRCVPFNES-VKTCEVAAWCPV
P2X1 QTQGHCAENPE--GGICQDDSGCTPGKAERKAQGIRTGNCVPFN-GTVKTCEIFGWCPV
P2X2 QTLGTCPESMRVHSSTCHSDDDCIAGQLDMQGNGIRTGHCVPYYHGDSKTCEVSAWCPV
P2X3 QMQGFCPENEEKYR--CVSDSQC--GPERFPGGGILTGRCVNYSS-VLRTCEIQGWCPT
P2X5 QRQGICAEREGIPDGECSEDDDCHAGESVVAGHGLKTGRCLRVGNSTRGTCEIFAWCPV
P2X6 QVQGRCPEHPSVPLANCWADADCPEGEMGTYSHGIKTGQCVPFNG-THRTCEIWSWCPV
P2X7 QEQKLCPEYPSRGKQ-CHSDQGCIKGWMDPQSKGIQTGRCIPYDQK-RKTCEIFAWCPA
          *          *     *                *          *     *
```

Shinozaki et al. Figure S4
